# A Well-Studied Disease-Resistance Gene Shows No Signs of Selection

**DOI:** 10.1371/journal.pbio.0030400

**Published:** 2005-11-01

When our ancestors switched from hunting and gathering to farming about 10,000 years ago, they unwittingly unleashed new selective pressures associated with different diets, increased population density, and novel infectious diseases. This period of rapid change, it is widely thought, also precipitated many genetic adaptations, some conferring resistance to disease. One gene presumed to have undergone positive selection has generated significant interest because of its role in HIV infection. The gene encodes a chemokine receptor called CCR5 on the surface of white blood cells that, along with CD4, mediates HIV entry into the cells.

In 1996, independent research groups discovered a 32–base pair deletion, called ▵32, in the gene's coding region that confers HIV resistance to individuals with two copies of the gene variant, or allele, and delayed AIDS progression to those with one copy. The mutation was relatively common among northern Europeans but virtually absent in non-Caucasians. In a 1998 study, researchers estimated the mutation's age roughly 700 years by analyzing the genetic variation patterns of 192 Caucasian chromosomes. Because it is unlikely for a young mutation to reach a high population frequency by chance alone, it was thought that some selective agent—alternately thought to be bubonic plague and smallpox—had accelerated its spread.

But now Pardis Sabeti, Eric Lander, and their colleagues report that the mutation may be much older than previously thought—and find no evidence of positive selection. The 1998 study based CCR5 ▵32's age, in part, on evidence that the allele was inherited along with two genomic markers, called microsatellites, positioned farther away than would be expected under neutral evolution. Higher than expected linkage disequilibrium (LD)—the distance between linked sequences— suggests the linked sequences are under positive selection. LD shrinks over time because the recombination that occurs during sperm and egg cell development reshuffles sequences around the mutation. Using recently available genome-wide sequence variation data, Sabeti et al. show that the LD and pattern of genetic variation at the allele's locus isn't unusual when compared to the rest of the genome.

For their study, Sabeti et al. analyzed the CCR5 ▵32 polymorphism, two microsatellites, and 70 single nucleotide polymorphisms (SNPs) on Chromosome 3, where the mutation resides, in 340 chromosomes from European, Chinese, and African (Yoruba, Nigeria) populations. They compared the genetic diversity at CCR5 to genomic regions from two large empirical datasets: 2,359 SNPs in 168 immunological genes, located across the genome studied in the same 340 chromosomes, and 63,149 SNPs on Chromosome 3 studied for the same populations from the International Haplotype Map Consortium project. The frequency with which CCR5 variants appeared within and between populations was not unusual compared to the 168 genes or to other regions of Chromosome 3. For the gene to be under selection, it should have shown either decreased or increased diversity within a population or greater variation in distribution among populations. As for CCR5 ▵32's higher frequency in European populations, that isn't unusual either, it turns out: many other polymorphisms found in similar frequencies (7%–9%) in Europeans do not occur in the other populations.

To estimate the LD around CCR5 ▵32, Sabeti et al. first applied the technique used in the 1998 study, and similarly found that chromosomes with the mutation have much longer LD than chromosomes without the mutation. But when the authors analyzed the full range of variation at the CCR5 locus, rather than simply examining ▵32 versus non-▵32 variation, they found two variants with longer LD than ▵32. They further compared the LD around CCR5 ▵32 to LD around other similarly prevalent mutations on Chromosome 3, and found that it was not unusual. Finally, Sabeti et al. used the new sequence variation data to remap the microsatellite markers, and showed that they're much closer to the mutation than previously thought, pushing the mutation's age back to roughly 5,000 years ago.

These results show that genetic variation around CCR5 ▵32 is not so different from the rest of the genome, and find no sign of recent selection for the allele. The absence of evidence is not evidence of absence, but the study raises the important issue that evidence for selection should now be examined and re-examined in a genome-wide context. As more genome-wide datasets become available, scientists will be able to compare the pattern of variation at every gene with overall genomic variation. And by finding the signs of selection in our genes, these tools can point to the evolutionary events that shaped our history and shed light on the genetic roots of disease resistance. —*Liza Gross*

